# Laser cavitation rheology for measurement of elastic moduli and failure strain within hydrogels

**DOI:** 10.1038/s41598-020-68621-y

**Published:** 2020-08-04

**Authors:** Justin C. Luo, Herman Ching, Bryce G. Wilson, Ali Mohraz, Elliot L. Botvinick, Vasan Venugopalan

**Affiliations:** 1 Department of Biomedical Engineering, University of California, Irvine, Irvine, CA 92697-2715 USA; 2 Beckman Laser Institute & Medical Clinic, University of California, Irvine, CA 92697-2575 USA; 3 Department of Chemical and Biomolecular Engineering, University of California, Irvine, 916 Engineering Tower, Irvine, CA 92697-2580 USA; 4 Present Address: Department of Biomedical Engineering, Cornell University, Ithaca, NY USA

**Keywords:** Engineering, Materials science, Optics and photonics, Physics, Cell signalling, Cellular imaging, Biopolymers in vivo, Biomaterials, Tissue engineering

## Abstract

We introduce laser cavitation rheology (LCR) as a minimally-invasive optical method to characterize mechanical properties within the interior of biological and synthetic aqueous soft materials at high strain-rates. We utilized time-resolved photography to measure cavitation bubble dynamics generated by the delivery of focused 500 ps duration laser radiation at λ = 532 nm within fibrin hydrogels at pulse energies of *E*_*p*_ = 12, 18 µJ and within polyethylene glycol (600) diacrylate (PEG (600) DA) hydrogels at *E*_*p*_ = 2, 5, 12 µJ. Elastic moduli and failure strains of fibrin and PEG (600) DA hydrogels were calculated from these measurements by determining parameter values which provide the best fit of the measured data to a theoretical model of cavitation bubble dynamics in a Neo-Hookean viscoelastic medium subject to material failure. We demonstrate the use of this method to retrieve the local, interior elastic modulus of these hydrogels and both the radial and circumferential failure strains.

## Introduction

Our motivation in the development of laser cavitation rheology (LCR) is to establish the capability to non-invasively measure the mechanical properties of the three-dimensional (3D) environment in which biological cells reside and interact with both other cells and the extracellular matrix (ECM)^1,2^. The ECM is most often a soft material that is synthesized and assembled by the resident cells^3,4^. Cells adhere to this structural scaffold and establish a niche microenvironment comprised of both biochemical and mechanical cues^5–9^. Our current understanding of cellular signalling due to mechanical forces (mechanotransduction), and the influence of mechanical forces on biological processes (mechanobiology) is based largely on studies performed in two-dimensional (2D) culture where cells are plated on either plastic or glass surfaces coated with adhesion proteins. Yet, cell cultivation on flat substrates often does not recapitulate the true physiological context. As a result, 3D culture systems have emerged wherein cells are typically enclosed in viscoelastic hydrogels fabricated from ECM-derived materials in an attempt to mimic the cellular microenvironment in vivo^10–15^.

In this context, investigation of the mechanoreciprocity^16,17^ i.e., the interplay between local ECM stiffness^18,19^ and cellular mechanotransduction would benefit from a minimally-invasive method that can mechanically stimulate and measure the local viscoelastic response of soft biological materials^20^. In addition, there is a growing awareness of the role of mechanoreciprocity in the origin and development of disease processes including cancer invasion and metastasis, chronic wounds, hearing loss, and osteoporosis^21^. Moreover, the development of assays, which can be easily integrated within a conventional biological microscopy or cell cytometry system, with the ability to measure changes in mechanical properties due to ECM remodelling may prove invaluable to understanding underlying biological disease processes and can serve as a means to screen potential therapeutic compounds^20^.

Techniques that are frequently employed for mechanical characterization of soft materials include rheometry^22–24^, atomic force microscopy^25–28^ (AFM), passive microrheology^29,30^, and active microrheology^31–37^. However, neither rheometry nor AFM provide a direct means to probe the local interior of a bulk material. While microrheology is capable of probing the internal properties of soft matter at the microscale level, this method requires the introduction of exogenous particles. None of these techniques are capable of characterizing soft materials at high frequencies or large strain-rates. Recent reports have introduced techniques for determining the internal mechanical characteristics of soft materials using cavitation bubbles initiated by syringe needles^38–40^, acoustics^41,42^, and pulsed lasers^43^. While these methods provide alternate means to characterize soft materials, they suffer from one or more of the following limitations: (a) the need for probe insertion into the material, (b) inability to control the position at which the material is tested or (c) lack of consideration of material failure in the measurement process. Here we present our development of LCR to measure mechanical properties using focused pulsed laser microbeam radiation to generate small (< 200 µm radius) cavitation bubbles within the interior of a soft material. The subsequent cavitation bubble dynamics, which occur on timescales of < 30 µs, are measured and analysed to determine the sample’s local mechanical properties. The analysis used for LCR considers the bubble dynamics occurring within a viscoelastic material capable of undergoing material deformation with potential failure at high strains and strain rates.

## Approach

### Laser cavitation rheology platform

Our proposed LCR platform combines three elements: (a) impulsive deformation of soft materials using a single pulsed laser-generated microcavitation bubble, (b) measurement of cavitation bubble dynamics using time-resolved photography with automated image analysis, and (c) retrieval of the elastic modulus and failure strain through analysis of the measured cavitation bubble dynamics. The use of focused pulsed laser microbeam irradiation provides a controlled means to generate mesoscopic cavitation bubbles within the interior of hydrogels to impart finite material deformation. The frequency, magnitude, and spatial coverage of the imparted stresses and deformation can be modified by adjusting laser pulse energy and/or pulse duration^44–47,67^ which can enable materials characterization on varying length scales and strain-rates. We hypothesize that the (visco-)elastic material properties that influence bubble dynamics are retrievable through measurement of these dynamics and subsequent analysis that considers potential material failure. Using this approach, we examined the properties of fibrin and polyethylene glycol (600) diacrylate (PEG (600) DA) gels. Fibrin gels of 2.5 and 10 mg mL^−1^ concentration were irradiated using 500 ps duration laser microbeam pulses of energy *E*_*p*_ = 12, 18 µJ whereas 6% and 7% v/v PEG (600) DA hydrogels were examined utilizing pulse energies of *E*_*p*_ = 2, 5, 12 µJ.

## Materials and methods

### Hydrogel fabrication

Fibrinogen from human plasma (F3879, Sigma) was dissolved in Hank’s Balanced Salt Solution without the addition of either Ca^2+^ or Mg^2+^ ions (HBSS-) and phenol red (14175-095, Gibco) in a 37 °C water bath for 1 h. The fibrinogen solution was then sterilized using a 0.2 µm PES syringe filter. Polymerization into fibrin hydrogels was initiated following the administration of 1 U mL^−1^ thrombin from human plasma (T7009, Sigma) into the fibrinogen solution. Fibrin hydrogels were incubated at room temperature for 5 min, transferred to a 37 °C incubator for 25 min, hydrated with HBSS-, and incubated at 37 °C for an additional 2 h to ensure full polymerization. Polyethylene glycol (600) diacrylate (Sartomer) was diluted in HBSS-buffer and 0.5% by volume of the free radical photoinitiator Darocur 1173 (Ciba). PEG (600) DA hydrogels were photocured by exposing to UV (OmniCure S1000, EXFO) for 45 s.

### Time-resolved cavitation imaging setup

**Figure 1:**
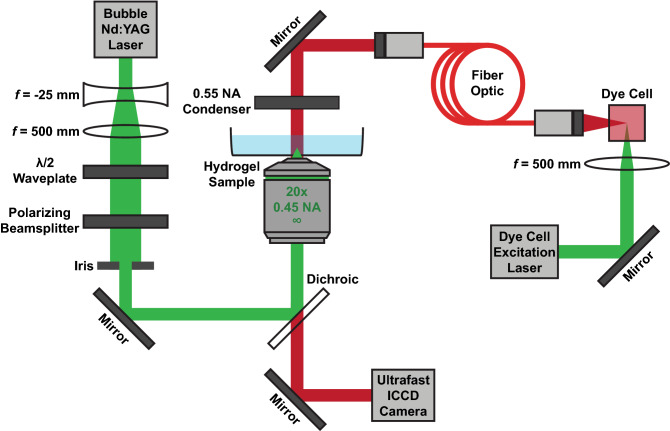
Laser-induced cavitation rheology setup. The laser microscope consists of a microchip laser emitting λ = 532 nm, 500 ps duration pulses integrated within a standard inverted microscope to enable the generation of cavitation bubbles within hydrogel samples. Cavitation dynamics were measured via time-resolved photography where bubbles were imaged using a high-speed ICCD camera with illumination provided by a laser-excited dye cell. Prepared using Adobe Illustrator CS6.

Figure 1 depicts the laser microscope setup we utilized to determine cavitation dynamics in the hydrogel interior via time-resolved photography. Cavitation was initiated by pulsed laser microbeam irradiation of the hydrogel using 500 ps duration pulses at λ = 532 nm with pulse energies of 2–18 µJ provided by a Q-switched pulsed microchip laser (PNG-M03012, Teem Photonics). We utilized a negative/positive lens pair with focal lengths of *f* = − 25 mm and *f* = 500 mm to expand and collimate the laser beam prior to introduction into the microscope. Pulse energy was adjusted via a rotatable λ/2 wave-plate and polarizing beam splitter. An iris cropped the peripheral regions of the laser beam to allow the 8 mm diameter central portion to enter the right port of an inverted microscope (IX-81, Olympus). The laser was directed by a dichroic mirror (ZT532NBDC, Chroma) to the rear aperture of a 20 × 0.45 NA objective (LUCPlanFLN, Olympus), which delivered a focused microbeam into the hydrogel interior.

Cavitation dynamics were determined using a gated intensified charge-coupled device (ICCD) camera (4-Picos, Stanford Computer Optics) with a 5 ns exposure time. Fluorescence emission from a dye cell (LDS 698, Exciton) pumped by a separate λ = 532 nm frequency-doubled Q-switched Nd:YAG laser (Brilliant B, Quantel) provided imaging illumination, which was collected and directed to the microscope condenser via fiber optics. Temporal control for the delivery of the pulsed laser microbeam, image illumination, and camera gate was achieved using a delay generator (Model 575, Berkeley Nucleonics Corporation). Electronic signals were examined on an oscilloscope (WaveRunner 6051A, LeCroy) to synchronize the camera gate and image illumination relative to the laser microbeam irradiation.

Cavitation bubbles were generated at a height of 550 µm and 10 µm above the glass surface for fibrin and PEG (600) DA hydrogel samples, respectively. A single full bubble oscillation cycle comprising of bubble formation, expansion, and collapse was characterized in all hydrogel experiments. Our time-resolved photography setup is only capable of obtaining a single image for each cavitation bubble event. Thus, for a fixed set of laser irradiation parameters, we assume that the cavitation formation process is reproducible and capture the entire bubble growth/collapse cycle by obtaining images at different time delays corresponding to irradiation at different spatial locations within the hydrogel. In all cavitation experiments, at least five data points were recorded per delay time in 1 µs increments.

### Image analysis to retrieve cavitation bubble size

An automated image analysis scheme was implemented to enable quantitative measurement of the cavitation bubble dynamics using a MATLAB (MathWorks) script. All images were segmented using *k*-means clustering with two classes where we calculated an equivalent radius from the segmented pixel area by assuming spherical symmetry of the cavitation bubble cross-section.

### Modelling of bubble dynamics in a viscoelastic medium

We start by adopting the approach developed by Gaudron et al.^48^ to formulate the governing equation for cavitation bubble dynamics in a Neo-Hookean viscoelastic medium:

1 $$R_{B} \frac{{d^{2} R_{B} }}{{dt^{2} }} + \frac{3}{2}\left( {\frac{{dR_{B} }}{dt}} \right)^{2} = \frac{{p_{B} - p_{\infty } }}{\rho } - \frac{2S}{{\rho R_{B} }} - \frac{4\nu }{{R_{B} }}\frac{{dR_{B} }}{dt} - \frac{E}{\rho },$$

where *R*_*B*_ is the cavitation bubble radius which progresses with time *t*, *ν* the kinematic viscosity of the surrounding material, *p*_*B*_ the gas pressure inside the bubble, *p*_∞_ the surrounding isotropic pressure far from the bubble, *S* the surface tension, *ρ* the material density, and *E* represents the Neo-Hookean elastic stress that the surrounding material imposes onto the bubble surface given by:

2 $$E=\frac{\eta }{2}\left[5-4\left(\frac{{R}_{0}}{{R}_{B}}\right)-{\left(\frac{{R}_{0}}{{R}_{B}}\right)}^{4}\right],$$

where *η* is the elastic modulus of the surrounding material and *R*_0_ is the equilibrium bubble radius. Here, the equilibrium bubble radius denotes the size of cavity before stress is applied to the external material and is calculated as *R*_0_ = *R*_max_(*p*_v_/*p*_∞_)^1/3κ^, where *p*_v_ (= 3,169 Pa for water at 25 °C) is the vapor pressure within the bubble and κ is the adiabatic index.

It is worth restating that our approach parallels that presented by Gaudron et al. Namely, the classic Rayleigh-Plesset model is modified for viscoelastic materials. The model focuses on mechanical effects using a non-linear neo-Hookean formalism and assumes thermal and mass transfer effects to be negligible. These latter assumptions are reasonable for instances such as ours, where inertial forces dominate the bubble dynamics^49^. Moreover, the large strains produced by inertial cavitation is properly modelled using a continuum mechanics finite strain formulation coupled with a neo-Hookean treatment of non-linear stress–strain behaviour at large deformations. The internal bubble pressure *p*_B_ is treated as an ideal gas with an internal pressure equivalent to the vapor pressure *p*_v_ at the maximum bubble radius *R*_max_, thus the internal bubble pressure *p*_B_ for any given bubble radius *R*_B_ is given by^50^: *p*_B_(*R*_B_) = *p*_v_(*R*_max_/*R*_B_)^3κ^.

### Incorporation of material failure

**Figure 2:**
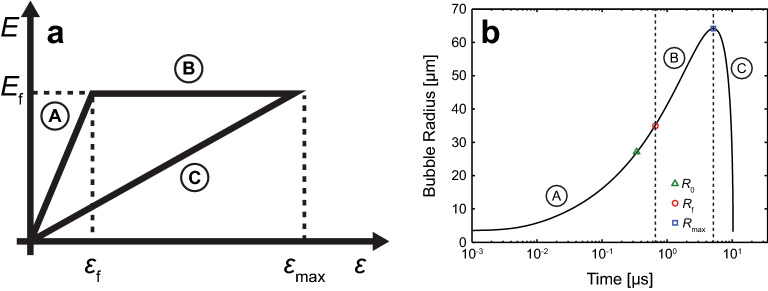
Bubble model constitutive relations. Depiction of the stress–strain relationship used to model viscoelastic material failure. (**a**) Stress–strain relationship utilized for modelling the elastic to plastic transition in the viscoelastic material. Adapted from Ref.^51^. (**b**) Representative cavitation dynamics diagram depicting the transition states for the viscoelastic material response which involves switching between constitutive relations at specific time points during the bubble cycle. Bubble dynamics are initially computed for an intact viscoelastic material which subsequently ruptures upon reaching the failure strain *ε*_*f*_ and bubble radius *R*_*f*_. Beyond the maximum bubble radius *R*_*max*_, cavitation dynamics in the recovery phase are evaluated. The letters correspond to behaviour of the material under deformation where: A—linear elastic, B—plastic deformation, and C—recovery. Prepared using MATLAB R2019b and Adobe Illustrator CS6.

Unfortunately, Eqs. (1) and (2) do not account for potential mechanical failure of the viscoelastic material produced by the expanding bubble. To account for this possibility, we modify the Neo-Hookean elastic stress term *E* based on a stress–strain relationship suggested by Glinsky et al.^51^ which was subsequently applied to cavitation bubble dynamics in hydrogels by Brujan and Vogel^52^ and depicted in Fig. 2a, where *ε*_f_, and *E*_f_ are used to denote the failure strain and stress of the material, respectively.

The stress–strain relationship depicted by path (A) represents the process whereby the bubble is expanding and the material has yet to fail. During this stage, we assume a linear relationship between elastic stress and deformation, and implement the unchanged Neo-Hookean constitutive equation shown in Eq. (2) now denoted as *E*_*L*_. Upon reaching a critical cavitation bubble radius or radial/circumferential strain, the material ruptures, as indicated by path (B) in the stress–strain curve. We model the stress associated with the resultant plastic deformation following failure using *E*_*f*_ given by^48^:

3 $${E}_{f}=\frac{\eta }{2}\left[5-4\left(\frac{{R}_{0}}{{R}_{f}}\right)-{\left(\frac{{R}_{0}}{{R}_{f}}\right)}^{4}\right],$$

where *R*_*f*_ represents the bubble radius at which the material fails. Once the bubble has reached its maximum size and begins to collapse, we model the elastic stress during recovery using *E*_*R*_ as represented by path (C) in Fig. 2a^48,51,52^:

4 $${E}_{R}=\frac{{E}_{f}\left[5-4\left(\frac{{R}_{0}}{{R}_{B}}\right)-{\left(\frac{{R}_{0}}{{R}_{B}}\right)}^{4}\right]}{\left[5-4\left(\frac{{R}_{0}}{{R}_{max}}\right)-{\left(\frac{{R}_{0}}{{R}_{max}}\right)}^{4}\right]}.$$

The implementation of this approach requires switching between these variations in the constitutive equation during the cavitation bubble cycle as illustrated in Fig. 2b. This requires an expression that relates the instantaneous bubble radius ${R}_{B}$ with the material strain, which is obtained by evaluating the Green-Lagrangian finite strain tensor at the bubble wall^54^:

5 $${\varepsilon }_{w,rr}=-\frac{1}{2}\left[{\left(\frac{{R}_{0}}{{R}_{B}}\right)}^{4}-1\right],$$

6 $${\varepsilon }_{w,\theta \theta }={\varepsilon }_{w,\varphi \varphi }=\frac{1}{2}\left[{\left(\frac{{R}_{B}}{{R}_{0}}\right)}^{2}-1\right],$$

where *ε*_*w,rr*_ and *ε*_*w,θθ*_ are the radial and circumferential strain at the bubble wall respectively. Note the negative sign in Eq. (5) is included simply to allow the compressive strain to adopt a positive value. We can use Eqs. (5) and (6) to determine the radial and circumferential failure strain of the material *ε*_*f,rr*_ and *ε*_*f,θθ*_, once the bubble radius at which the material fails *R*_*f*_ is determined. When *ε*_*w*_ < *ε*_*f*_, the linear elastic variation *E*_*L*_ is introduced into the elastic stress component *E*. The plastic deformation version *E*_*f*_ is implemented for *ε*_*f*_ < *ε*_*w*_ < *ε*_*max*_. The recovery term *E*_*R*_ is substituted upon reaching *ε*_*max*_ and for all times thereafter.

### Determination of elastic modulus in soft viscoelastic materials

We recover the mechanical properties of our hydrogel samples using a Levenberg–Marquardt algorithm that determines the parameters that provide a least-squares best fit between the experimental data and predictions provided by our cavitation bubble dynamics model. Specifically, the protocol involves the selection of initial values (guesses) for *R*_*max*_, $\eta$, and *ε*_*f,rr*_, which are parameters in the viscoelastic bubble model. The optimization process then determines values for maximum bubble radius *R*_*max*_, elastic modulus *η* and radial failure strain *ε*_*f,rr*_.

## Results

### Visualization of cavitation bubble dynamics in hydrogels

**Figure 3:**
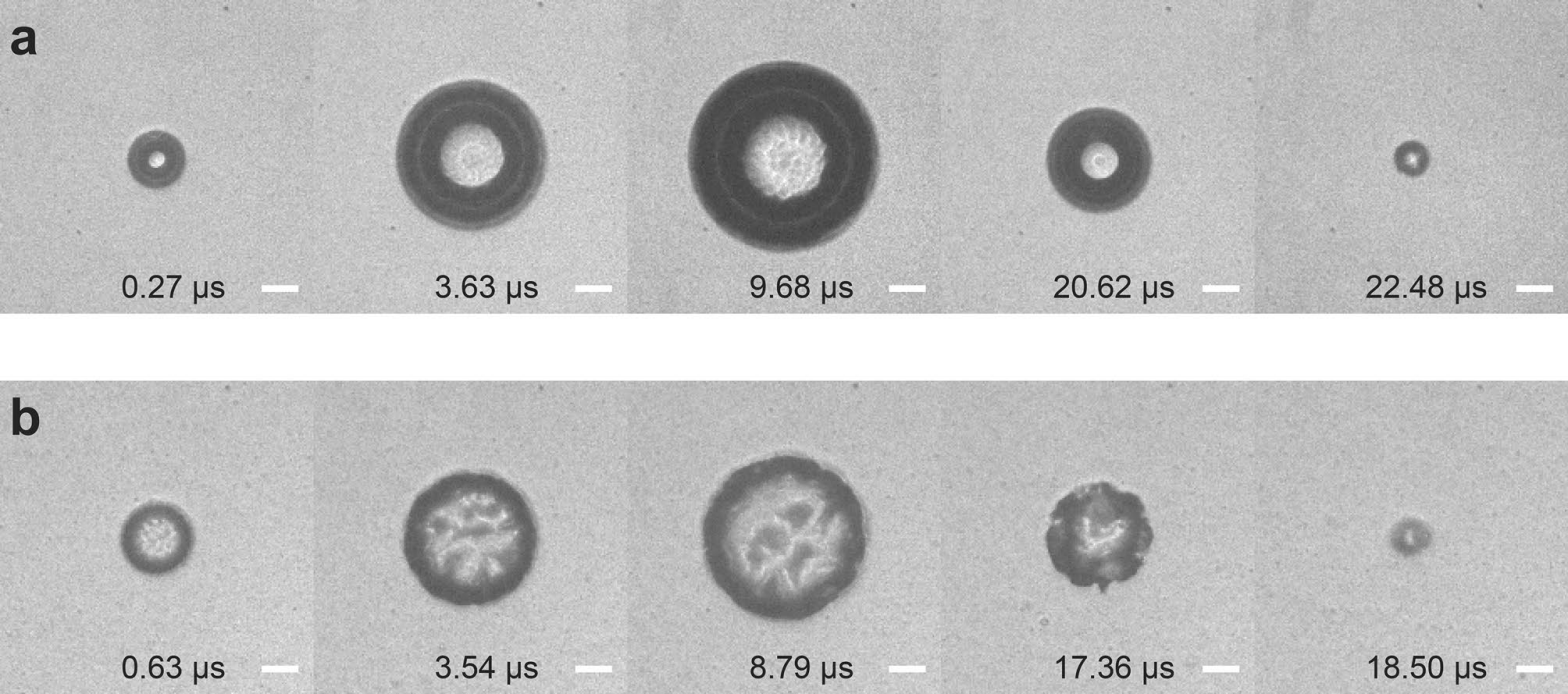
Cavitation bubble dynamics. Images of cavitation produced by pulsed laser irradiation at distinct time points within (**a**) 2.5 mg mL^−1^ fibrin hydrogels using a *E*_*p*_ = 12 µJ laser pulse with *R*_*max*_ = 126 µm and (**b**) 6% PEG (600) DA hydrogels from a *E*_*p*_ = 5 µJ laser pulse with *R*_*max*_ = 112 µm. Scale bar = 50 µm.

Visualization of the cavitation bubble dynamics obtained from time-resolved photography when formed within fibrin and polyethylene glycol (600) diacrylate (PEG (600) DA) hydrogels is depicted in Fig. 3a,b, respectively. A single full cavitation cycle consisting of bubble initiation, growth, and collapse produced in both hydrogel types occurred within tens of microseconds. The bubble size and cycle duration can be readily altered by adjusting laser pulse energy which enables delivery of variable strain-rates for probing the hydrogel interior.

### Quantification of cavitation bubble dynamics in hydrogels

**Figure 4:**
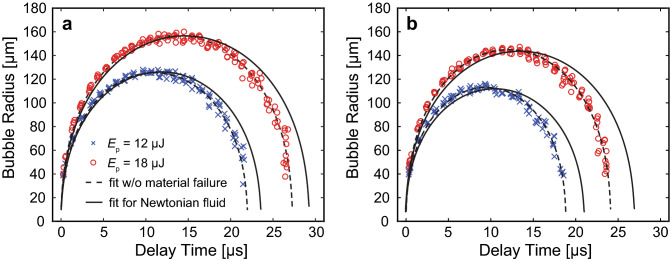
Cavitation dynamics in fibrin gels. Bubble dynamics in (**a**) 2.5 mg mL^−1^ and (**b**) 10.0 mg mL^−1^ fibrin hydrogels. × and ○ symbols represent the bubble dynamics generated using laser microbeam pulse energies of *E*_*p*_ = 12 µJ and 18 µJ, respectively. Solid and dashed lines represent predictions of a cavitation bubble dynamics model for a Newtonian fluid and a viscoelastic material without consideration of material failure, respectively. Prepared using MATLAB R2019b and Adobe Illustrator CS6.

In Fig. 4a,b, we plot the measured bubble dynamics within fibrin gels for laser pulse energies of *E*_*p*_ = 12 and 18 µJ at fibrin concentrations of 2.5 and 10.0 mg mL^−1^, respectively, and compare them to a theoretical prediction of cavitation bubble dynamics which considers the viscoelastic nature of the material but does not consider material failure. For a fixed laser pulse energy, we observed reductions in the maximum bubble radius and duration of the cavitation bubble cycle with increasing concentration of fibrin. As a point of comparison, identical bubble sizes formed in water, which represents a material with similar density but no elasticity, would have cavitation cycle times of 22.9 and 28.8 µs for the bubble sizes formed in the 2.5 mg mL^−1^ fibrin hydrogel at *E*_*p*_ = 12 µJ and 18 µJ, respectively, and 20.7 and 26.0 µs for the bubble sizes formed in the 10.0 mg mL^−1^ fibrin hydrogel at *E*_*p*_ = 12 µJ and 18 µJ, respectively. Thus the presence of elasticity shortens the cavitation bubble cycle times relative to bubbles of the same size formed in water. Specifically, for the data sets shown in Fig. 4, the ratio of the cavitation bubble cycle time in the hydrogel (*T*_hg_) to that in water (*T*_w_) is 0.933 at both pulse energies in the 2.5 mg mL^−1^ fibrin hydrogel and 0.894 in the 10 mg mL^−1^ fibrin hydrogel. Our observations are consistent with other studies and evidence that the elasticity of the fibrin gels offers resistance to the bubble dynamics^52,53^. Regardless, we find that a viscoelastic bubble model without consideration of material failure provides reasonable fits to the experimental data.

**Figure 5:**
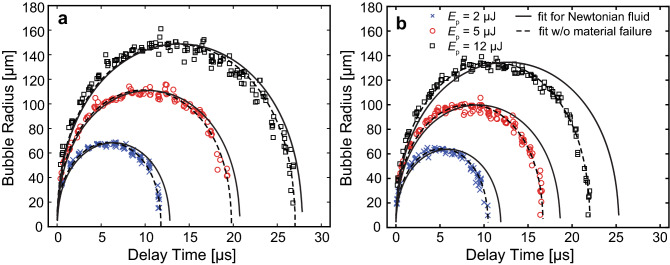
Bubble dynamics in PEG (600) DA hydrogels. Cavitation bubble dynamics measured in (**a**) 6% and (**b**) 7% PEG (600) DA hydrogels. ×, ○ and □ symbols represent the bubble dynamics measured using pulse energies of at *E*_*p*_ = 2 µJ, 5 µJ, and 12 µJ, respectively. Solid and dashed lines represent predictions of a cavitation bubble dynamics model for a Newtonian fluid and a viscoelastic material without consideration of material failure, respectively. Prepared using MATLAB R2019b and Adobe Illustrator CS6.

 Figure 5 provides the measured bubble dynamics in 6 and 7% PEG (600) DA hydrogels using laser pulse energies of *E*_*p*_ = 2, 5, and 12 µJ. We observed that cavitation dynamics in PEG (600) DA hydrogels behaved similarly to those measured in fibrin gels, along with model predictions which provided fits of comparable quality to the experimental data when material failure is not considered. Again, for a fixed laser pulse energy, we observed reductions in the maximum bubble radius and duration of the cavitation bubble cycle with increasing PEG concentration. As a point of comparison, identical bubble sizes formed in water, which represents a material with similar density but no elasticity, would have cavitation cycle times of 12.6, 19.8, and 27.9 µs for the bubble sizes formed in the 6% PEG hydrogel at *E*_*p*_ = 2, 5, and 12 µJ, respectively, and 12.0, 18.3, and 24.4 µs for the bubble sizes formed in the 7% PEG hydrogel at *E*_*p*_ = 2, 5, and 12 µJ, respectively. Thus, the presence of elasticity shortens the cavitation bubble cycle times relative to bubbles of the same size formed in water. Specifically, for the data sets shown in Fig. 5, the ratio of the cavitation bubble cycle time in the hydrogel (*T*_hg_) to that in water (*T*_w_) lies in the range 0.922–0.972 for the 6% PEG hydrogel and 0.758–0.878 in the 7% PEG hydrogel.

**Figure 6:**
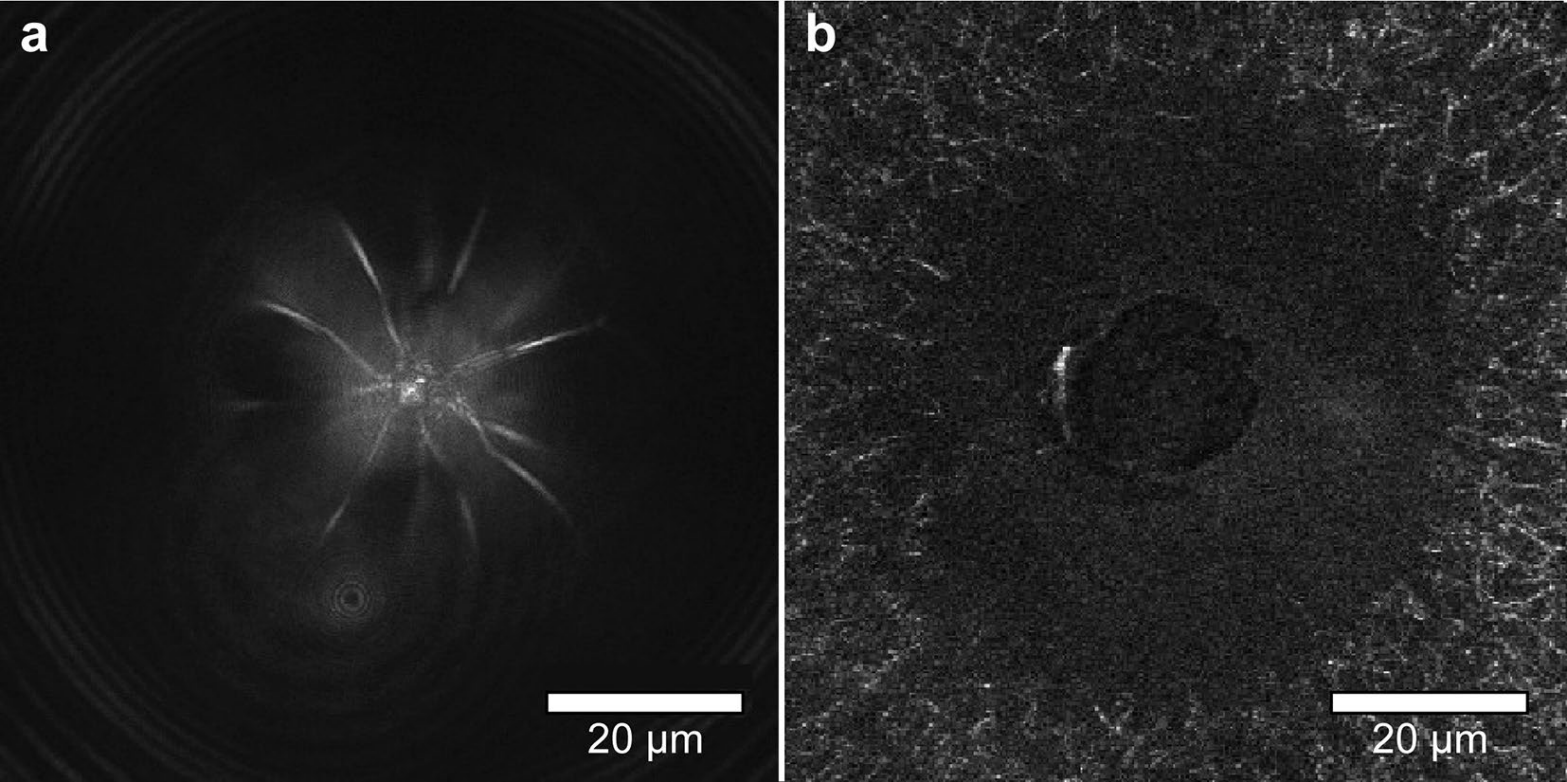
Hydrogel rupture created by LCR. Material failure produced by a single laser generated cavitation bubble produced within a (**a**) 6% PEG (600) DA and (**b**) 2.5 mg mL^−1^ fibrin hydrogel as revealed by laser-scanning reflectance confocal microscopy. Both samples were irradiated by a single *E*_*p*_ = 12 µJ laser pulse resulting in cavitation bubble formation and subsequent material failure. Scale Bar = 20 µm.

Table 1 shows the parameter values recovered from the fit of the experimental data to predictions provided by the bubble model. The general trends for the elasticity values look reasonable, with higher concentrations of the fibrin and PEG (600) DA hydrogels leading to larger elastic moduli. However, material damage within both fibrin and PEG (600) DA gels were observed following the initiation of bubbles as visualized by laser scanning reflectance confocal microscopy images shown in Fig. 6. This affirms our hypothesis that the radial compression and circumferential tension produced by the laser-initiated cavitation bubbles can lead to material failure. Moreover, this suggests that incorporation of material failure within our cavitation bubble model is essential to provide a credible analysis of the cavitation bubble dynamics data. Such failure determined from data acquired in a single cavitation bubble cycle is not considered in the current literature^41,43^.

**Table 1:** LCR results for hydrogel samples using viscoelastic bubble model without material failure.

Material	Sample #	Pulse energy *E*_*p*_ (µJ)	Maximum bubble radius *R*_*max*_ (µm)	Elastic modulus *η* (kPa)
2.5 mg mL^−1^ Fibrin	3	12	124 ± 3	7.0 ± 0.6
	2	18	156 ± 2	8.1 ± 0.1
10.0 mg mL^−1^ Fibrin	5	12	112 ± 2	13 ± 1
	5	18	141 ± 3	12 ± 2
6% PEG (600) DA	4	2	68 ± 1	8 ± 4
	3	5	107 ± 4	7 ± 1
	3	12	151 ± 2	5 ± 2
7% PEG (600) DA	4	2	65 ± 1	16 ± 3
	3	5	99 ± 1	11 ± 2
	5	12	132 ± 3	15 ± 2

Values for the maximum bubble radius *R*_*max*_ and elastic modulus as determined from fitting our measurements of the laser-microbeam generated cavitation bubble dynamics with our viscoelastic bubble model without consideration of material failure. Values reported are mean ± standard deviation.

### Effect of elastic modulus and failure strain on cavitation bubble dynamics in hydrogels

**Figure 7:**
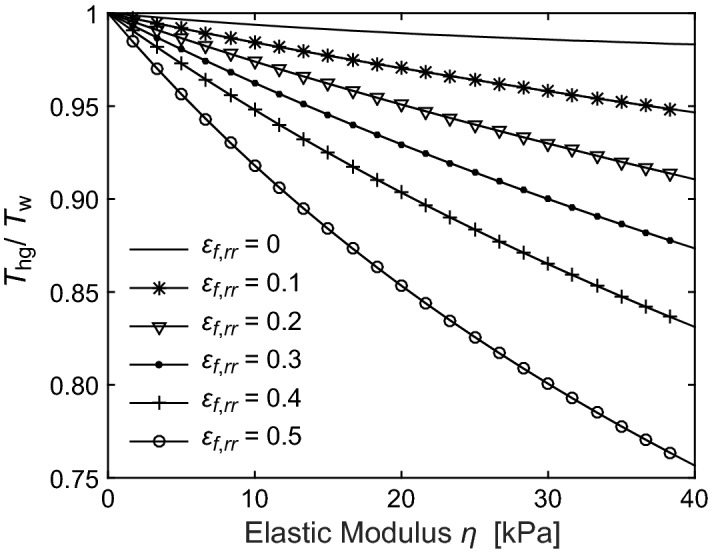
Shortening of cavitation bubble oscillation time as a function of elastic modulus and failure strain. Cavitation bubble dynamics for fixed maximum bubble size (*R*_*max*_ = 120 µm) for variable elastic modulus (*η* = 0–40 kPa) and radial failure strain *ε*_*f,rr*_ (0–0.5 in increments of 0.1). Prepared using MATLAB R2019b and Adobe Illustrator CS6.

Visual examination of the hydrogels, which indicates material failure following the application of our LCR method, compelled us to implement constitutive relations that explicitly accommodate material failure. To demonstrate the sensitivity of bubble dynamics to both the elastic modulus *η* and material failure strain *ε*_*f*_, in Fig. 7 we provide predictions for the shortening of the bubble oscillation time (i.e. the ratio of the bubble oscillation time, *T*_*hg*_*,* relative to that in a material with no elasticity, *T*_*w*_) as a function of both elastic modulus and radial failure strain. As expected, we find that the bubble cycle time shortens with increasing elastic modulus of the material as well as radial failure strain. This behaviour is intuitive as a larger failure strain provides an increased elastic restoring force prior to material failure, which results in a shorter oscillation time.

### Recovery of elastic modulus and failure strain from measured cavitation bubble dynamics in hydrogels

**Figure 8:**
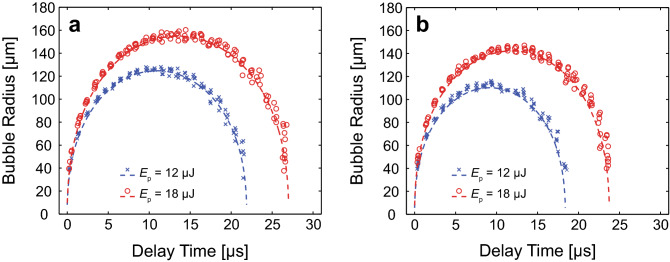
Model fits to measured cavitation bubble dynamics in fibrin gels. Model-predicted cavitation bubble dynamics using the best parameter fits and experimental data points in fibrin hydrogels. (**a**) 2.5 mg mL^−1^ fibrin gel with model fits generated for pulse energies *E*_*p*_ = 12 µJ (*R*_*max*_ = 126 µm, *η* = 20 kPa, *ε*_*f,rr*_ = 0.25, *ε*_*f,θθ*_ = 0.21) and *E*_*p*_ = 18 µJ (*R*_*max*_ = 156 µm, *η* = 23 kPa, *ε*_*f,rr*_ = 0.25, *ε*_*f,θθ*_ = 0.20). (**b**) 10 mg mL^−1^ fibrin gel model predictions estimated from *E*_*p*_ = 12 µJ (*R*_*max*_ = 113 µm, *η* = 42 kPa, *ε*_*f,rr*_ = 0.23, *ε*_*f,θθ*_ = 0.18) and *E*_*p*_ = 18 µJ (*R*_*max*_ = 145 µm,* η* = 36 kPa, *ε*_*f,rr*_ = 0.27, *ε*_*f,θθ*_ = 0.23). The bubble dynamics model predictions are shown by the dashed curves while the experimental data are shown by the symbols. ○ and × symbols correspond to cavitation dynamics produced by laser pulse energies of *E*_*p*_ = 12 and 18 µJ, respectively. Prepared using MATLAB R2019b and Adobe Illustrator CS6.

Figure 8a,b, provide fits to typical experimental data sets of the measured cavitation bubble dynamics in 2.5 and 10 mg mL^−1^ fibrin hydrogels, respectively. The recovered parameter values of maximum bubble radius *R*_*max*_, elastic modulus *η*, and both radial and circumferential failure strains *ε*_*f,rr*_ and *ε*_*f,θθ*_ are provided in the caption for the specific data sets shown. These graphs establish that the cavitation bubble dynamics calculated using the retrieved parameter values conforms well to the experimental measurements in fibrin hydrogels.

Table 2 provides the recovered parameter values for maximum bubble radius *R*_*max*_, elastic modulus *η*, and both radial and circumferential failure strains *ε*_*f,rr*_ and *ε*_*f,θθ*_ that provide a best fit between our model and experimental measurements of bubble dynamics in our fibrin hydrogels. As expected, we find that *R*_*max*_ diminishes when fibrin gel concentration is increased from 2.5 to 10.0 mg mL^−1^ for a fixed pulse energy. The recovered parameter values show an expected increase in *η* with increasing fibrin gel concentration. We also find that the predicted radial and circumferential failure strains for the fibrin material are relatively uniform across concentration and pulse energies.

**Table 2:** LCR results for fibrin gel samples with explicit consideration of material failure.

Fibrin concentration	Averaged Sample #	*E*_*p*_ (µJ)	*R*_*max*_ (µm)	*η* (kPa)	*ε*_*f,rr*_ (–)	*ε*_*f,θθ*_ (–)
2.5 mg mL^−1^	3	12	124 ± 3	25 ± 7	0.21 ± 0.05	0.16 ± 0.05
	2	18	155 ± 2	23.6 ± 0.7	0.24 ± 0.01	0.19 ± 0.02
10.0 mg mL^−1^	5	12	112 ± 2	41 ± 10	0.24 ± 0.05	0.20 ± 0.07
	5	18	142 ± 3	37 ± 4	0.26 ± 0.03	0.22 ± 0.04

Values for the maximum bubble radius *R*_*max*_, elastic modulus *η*, radial failure strain *ε*_*f,rr*_, and circumferential failure strain *ε*_*f,θθ*_ determined from our measurements of the laser-microbeam generated cavitation bubble dynamics in 2.5 and 10 mg mL^−1^ fibrin gels in conjunction with our optimization algorithm. Values reported are mean ± standard deviation.

**Figure 9:**
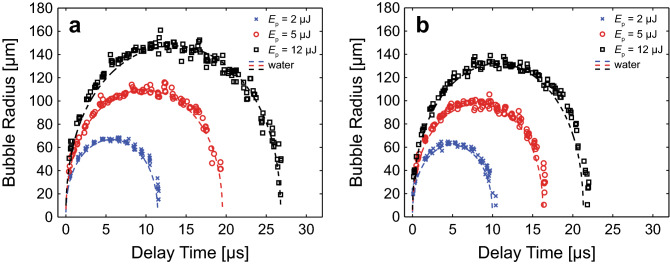
Model fits to measured cavitation bubble dynamics in PEG (600) DA hydrogels. Model-predicted cavitation bubble dynamics using the best parameter fits and experimental data points in PEG (600) DA hydrogels. (**a**) 6% PEG (600) DA gel model fits predicted by *E*_*p*_ = 2 µJ (*R*_*max*_ = 69 µm, *η* = 27 kPa, *ε*_*f,rr*_ = 0.24, *ε*_*f,θθ*_ = 0.2), *E*_*p*_ = 5 µJ (*R*_*max*_ = 111 µm, *η* = 22 kPa, *ε*_*f,rr*_ = 0.14, *ε*_*f,θθ*_ = 0.09), and *E*_*p*_ = 12 µJ (*R*_*max*_ = 149 µm, *η* = 20 kPa, *ε*_*f,rr*_ = 0.12, *ε*_*f,θθ*_ = 0.07) and (**b**) 7% PEG (600) DA hydrogel model predictions evaluated from *E*_*p*_ = 2 µJ (*R*_*max*_ = 64 µm, *η* = 32 kPa, *ε*_*f,rr*_ = 0.37, *ε*_*f,θθ*_ = 0.5), *E*_*p*_ = 5 µJ (*R*_*max*_ = 100 µm, *η* = 39 kPa, *ε*_*f,rr*_ = 0.24, *ε*_*f,θθ*_ = 0.19), and *E*_*p*_ = 12 µJ (*R*_*max*_ = 135 µm, *η* = 50 kPa, *ε*_*f,rr*_ = 0.25, *ε*_*f,θθ*_ = 0.20). The bubble dynamics model predictions are shown by the dashed curves while the experimental data are shown by the symbols. ○, ×, and □ symbols correspond to cavitation dynamics produced by laser pulse energies of *E*_*p*_ = 2, 5, and 12 µJ, respectively. Prepared using MATLAB R2019b and Adobe Illustrator CS6.

Figure 9a, b, provide typical experimental data sets for the measured cavitation bubble dynamics in 6% and 7% PEG (600) DA hydrogels, respectively. These figures also provide predictions of our bubble model at the parameter values that provide the best fit to the experimental data. The recovered parameter values of maximum bubble radius *R*_*max*_, elastic modulus *η*, and both radial and circumferential failure strains *ε*_*f,rr*_ and *ε*_*f,θθ*_ are provided in the caption for the specific data sets shown. These graphs establish that the calculated cavitation bubble dynamics in the PEG (600) DA hydrogels conform well to the experimental measurements.

Table 3 provides the recovered parameter values for maximum bubble radius *R*_*max*_, elastic modulus $\eta$, and both radial and circumferential failure strains *ε*_*f,rr*_ and *ε*_*f,θθ*_ that provide a best fit between our model and experimental measurements of bubble dynamics in our PEG (600) DA hydrogels. Similar to the fibrin results, for a fixed laser pulse energy, we recovered decreasing *R*_*max*_ values with increasing PEG (600) DA concentrations. The recovered *η* values show an increased stiffness of the PEG (600) DA gels with increasing material concentration from 6 to 7%. Except for the cases where 6% PEG (600) DA gels were irradiated with 5 and 12 µJ laser microbeam pulses, the recovered radial failure strains are fairly consistent across concentration and pulse energies.

**Table 3:** LCR results for PEG (600) DA hydrogel samples.

PEG (600) DA Concentration	Averaged Sample #	*E*_*p*_ (µJ)	*R*_*max*_ (µm)	*η* (kPa)	*ε*_*f,rr*_ (–)	*ε*_*f,θθ*_ (–)
6%	4	2	68 ± 1	22 ± 7	0.30 ± 0.05	0.3 ± 0.1
	3	5	107 ± 4	19 ± 4	0.17 ± 0.03	0.11 ± 0.02
	3	12	151 ± 2	27 ± 12	0.15 ± 0.03	0.09 ± 0.03
7%	4	2	65 ± 1	36 ± 4	0.32 ± 0.06	0.4 ± 0.1
	3	5	99 ± 1	29 ± 8	0.27 ± 0.03	0.25 ± 0.03
	6	12	132 ± 3	42 ± 12	0.27 ± 0.06	0.3 ± 0.1

Recovered values of maximum bubble radius *R*_*max,*_ elastic modulus *η*, and both radial and circumferential failure strain *ε*_*f,rr*_ and *ε*_*f,θθ*_ determined from laser-microbeam generated cavitation bubble dynamics in 6% and 7% PEG (600) DA hydrogels. Values reported are mean ± standard deviation.

## Discussion

We have demonstrated Laser Cavitation Rheology (LCR) as a means to measure and analyse cavitation bubble dynamics formed by pulsed laser microbeam irradiation to non-invasively quantify the elastic and failure strain properties of hydrogels at high strain rates. Unlike similar efforts proposed by Estrada et al.^43^, our method utilizes cavitation bubbles that are substantially smaller (by a factor of 3–5×), relies solely on the analysis of the first cavitation bubble cycle formed by the laser microbeam irradiation, and explicitly considers the material failure caused by the irradiation process. The use of smaller cavitation bubbles and the reliance on data obtained from the first cavitation bubble cycle enables LCR to measure material properties on a more local spatial scale and eliminates the potential influence of evolving material properties during subsequent cycles of bubble expansion and collapse^41^. Moreover, we have deliberately avoided the use of an optical imaging path that is perpendicular to the optical path used for cavitation bubble formation. Instead, we implemented collinear optical paths for both cavitation bubble formation and imaging that maintains the performance of such measurements on smaller spatial scales. This configuration enables the integration of LCR within conventional biological microscopy or cell cytometry systems.

The criticality of incorporating material failure in LCR is established by the visualization of the bubble dynamics using time-resolved imaging, and hydrogel morphology using laser scanning reflectance confocal microscopy. Moreover, these images provide evidence that the mode of failure may be different for fibrin vs. PEG hydrogels. While the time-resolved images in Fig. 3 show that LCR produces spherical bubbles in both material systems, the bubbles formed in fibrin have a smooth interface with the hydrogel while those formed in PEG have an irregular interface. Moreover, the confocal microscopy images of material failure show a large, smooth spherical defect and displaced fibres in the fibrin hydrogel. This stands in contrast to the PEG hydrogels where failure results in a punctate, irregular defect characterized by several radial microcracks. Taken together, we can infer that the ductile fibrin hydrogel network is resilient relative to radial compression but fails due to the tensile circumferential stresses imparted by the bubble. In contrast, PEG hydrogel shows evidence of brittle fracture through radial crack formation.

Material failure is further substantiated via comparison of our results for the elastic modulus *η* provided in Table 1, where material failure is not considered, with the results shown in Tables 2 and 3, where material failure is explicitly accounted for. The results obtained in Table 1, where material failure is not considered, provides elastic modulus values that are lower by a factor of 2.5–3.8 as compared to those in Tables 2 and 3. The disparity between the estimates obtained by these two constitutive relations decreases for larger values of the failure strain. This behaviour is intuitive as a failed material will appear to have a lower elastic modulus than it actually possesses prior to failure and the tested material will behave like an intact material for a longer duration of the cavitation bubble cycle for larger values of the failure strain.

**Figure 10:**
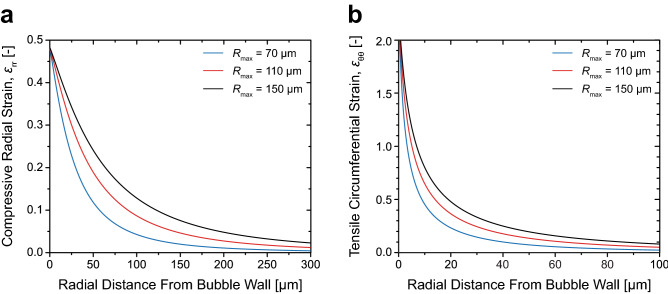
Radial and circumferential finite strain fields produced by laser microbeam generated cavitation bubbles. (**a**) Compressive radial strain and (**b**) tensile circumferential strain fields of the material points relative to the equilibrium bubble radius for various cavitation bubble sizes. Blue, red, and black curves denote the Green-Lagrangian finite strain fields plotted for bubble radii of *R*_max_ = 70, 110, and 150 µm, respectively. Corresponding values of R_0_ for these three cases are 29, 46, and 63 µm. Prepared using MATLAB R2019b and Adobe Illustrator CS6.

To further characterize the spatial extent and rate of material deformation, we utilize the Green-Lagrangian strain tensor^54^ to compute the magnitude of finite strains propagating throughout our viscoelastic hydrogels when deformed by cavitation expansion. Figure 10 depicts the spatial extent of radial and circumferential finite strain fields for material points relative to the equilibrium bubble radius for maximum bubble radii of *R*_*max*_ = 70, 110, and 150 µm. These strains are achieved in a duration of 5–15 µs resulting in strain rates of ~ 10^4^–10^5^ s^−1^. Not surprisingly, the radial extent of both the radial and circumferential strain fields extend further with increasing radial bubble size. Moreover, we find that material points immediately proximal to the bubble wall experience nearly maximal strains that decay rapidly with increasing radial distance. For cavitation bubbles with maximum radius *R*_*max*_ = 70–150 µm, the radial extent of material that experiences significant strain ranges from approximately 100–300 µm. Thus, the measures of elastic modulus that we obtain using LCR correspond to the mechanical integrity of the material on the mesoscopic (sub-millimetre) spatial scale.

To assess whether a continuum treatment of the material deformation is appropriate, we compute the mesh size *δ* of a crosslinked polymer using^55–57^

7 $$\delta = \left( {\frac{{k_{B} T}}{{G^{\prime}}}} \right)^{{{\raise0.7ex\hbox{$1$} \!\mathord{\left/ {\vphantom {1 3}}\right.\kern-\nulldelimiterspace} \!\lower0.7ex\hbox{$3$}}}}$$

where *k*_*B*_ is the Boltzmann constant, *T* is the absolute temperature, and *G*ʹ is the storage modulus, which we determined from conventional parallel-plate rheology (see Figs. S1 and S2 in Supplemental Information 1). Across fibrin and PEG (600) DA gels, the range of *G*ʹ values we determined span approximately 0.1–5 kPa, which equates to 9.4–34.5 nm for the characteristic pore size. The range of elastic moduli *η* recovered in fibrin and PEG (600) DA gels is approximately 19–42 kPa. As we showed above, the bubbles that we use in LCR effectively engages the material on a ~ 100 µm spatial scale that is much larger than the characteristic pore size. This supports the valid use of the continuum approach that we employed to treat material deformation and failure and that the results are not sensitive to the local microstructure immediately proximal to the bubble.

Our results establish the LCR platform for the non-invasive measurement of the physical properties of soft viscoelastic hydrogels at high strain-rates. This was achieved through precise measurement of bubble dynamics, formed within fibrin and PEG (600) DA gels that represent biological and synthetically-derived soft materials. We recovered hydrogel material characteristics from the experimental bubble dynamics data and demonstrated that LCR is capable of recovering the maximum cavitation bubble radius *R*_*max*_, elastic modulus *η*, and both radial and circumferential failure strains, *ε*_*f,rr*_ and *ε*_*f,θθ*_, of the material. LCR potentially offers new opportunities to investigate cellular mechanotransduction^47,67^ in a 3D context with simultaneous characterization of the ECM mechanical properties. Moreover, through its ability to subject soft materials to large deformations at high strain rates, LCR may provide a unique tool to study and probe fundamental rheological properties of soft materials on the mesoscopic scale under such extreme conditions^58^. Moreover, LCR is particularly useful to study and probe the rheological response of biological systems to impact/blast injury with potential utility in laser microsurgery^59,60^, molecular delivery^61^, cell lysis^62^, tissue ablation^63^, and traumatic brain or spinal cord injury^64–66^.

## Supplementary information

Supplementary information.
