# Supplementary material for: Laser cavitation rheology for measurement of elastic moduli and failure strain within hydrogels
Source: Sci Rep. 2020 Aug 4;10:13144. doi: 10.1038/s41598-020-68621-y (PMC7403306; doi:10.1038/s41598-020-68621-y)
Supplement: Supplementary file 1 — Supplementary information. [file 41598_2020_68621_MOESM1_ESM.pdf]

Supplemental Materials for

Laser Cavitation Rheology for Measurement of Elastic Moduli and Failure Strain within Hydrogels
Justin C. Luo, Herman Ching, Bryce G. Wilson, Ali Mohraz, Elliot L. Botvinick, and Vasan Venugopalan

Hydrogel Rheology Methods

To verify consistency of our material samples, rheological measurements were performed using stress-controlled rheometers. Fibrin gel rheology was performed on a rotational parallel-plate rheometer (MCR-301, Anton Paar). Following the addition of 1 U·mL^{-1} thrombin to fibrinogen, we quickly loaded the solution and lowered the parallel plate to initiate on-stage polymerization into fibrin gels. Fibrin samples were kept hydrated using a humidifying chamber on the MCR-301 rheometer. Rheology of PEG (600) DA hydrogels was conducted on a separate parallel-plate rheometer (DHR-3, TA Instruments) equipped with a borosilicate glass stage for *in-situ* photocuring. We similarly loaded PEG (600) DA mixed with 0.5% v/v of the photoinitiator Darocur 1173 onto the glass stage, lowered the parallel plate geometry, and *in situ* polymerization was initiated by 45 s of UV exposure. All fibrin and PEG (600) DA hydrogel tests were performed at room temperature using a 25 mm parallel plate geometry. Fibrin and PEG (600) DA samples were measured at a gap height of 200 µm and 300 µm, respectively. Hydrogel samples were not trimmed during loading to prevent pre-stressing and compromising the mechanical integrity. Amplitude sweeps ranging from 0.01–1000% strain were performed using a fixed frequency of 1 Hz to determine the linear viscoelastic regime. Frequency sweeps ranging from 0.01–100 Hz were carried out with amplitude set within the linear viscoelastic limit at 1% strain.

Macroscopic Rheology Data

To aid in the interpretation of these results, we chose to characterize the bulk properties of the fibrin and PEG (600) DA gels using conventional parallel plate rheometry. Rheometers measure the shear storage modulus G' whereas our proposed LCR technique determines the elastic modulus η. Shear deformation at various amplitudes and temporal frequencies were performed on these samples resulting in the data illustrated in Figure S1. As expected, the shear modulus G' increased as the fibrin concentration is increased from 2.5 to 10.0 mg·mL^{-1}. Such concentration dependent increases were also observed for the elastic modulus recovered by LCR. The amplitude sweeps indicate that fibrin fails approximately at a strain of 200–300% independent of our tested concentrations. Figure S2 depicts sweeps of frequency and amplitude for PEG (600) DA gels. Similarly, we also find that increasing PEG (600) DA concentrations from 6 to 7% led to stiffer hydrogels as indicated by greater values of G'. PEG (600) DA gels were also discovered to experience material failure in shear at values of near 100%.

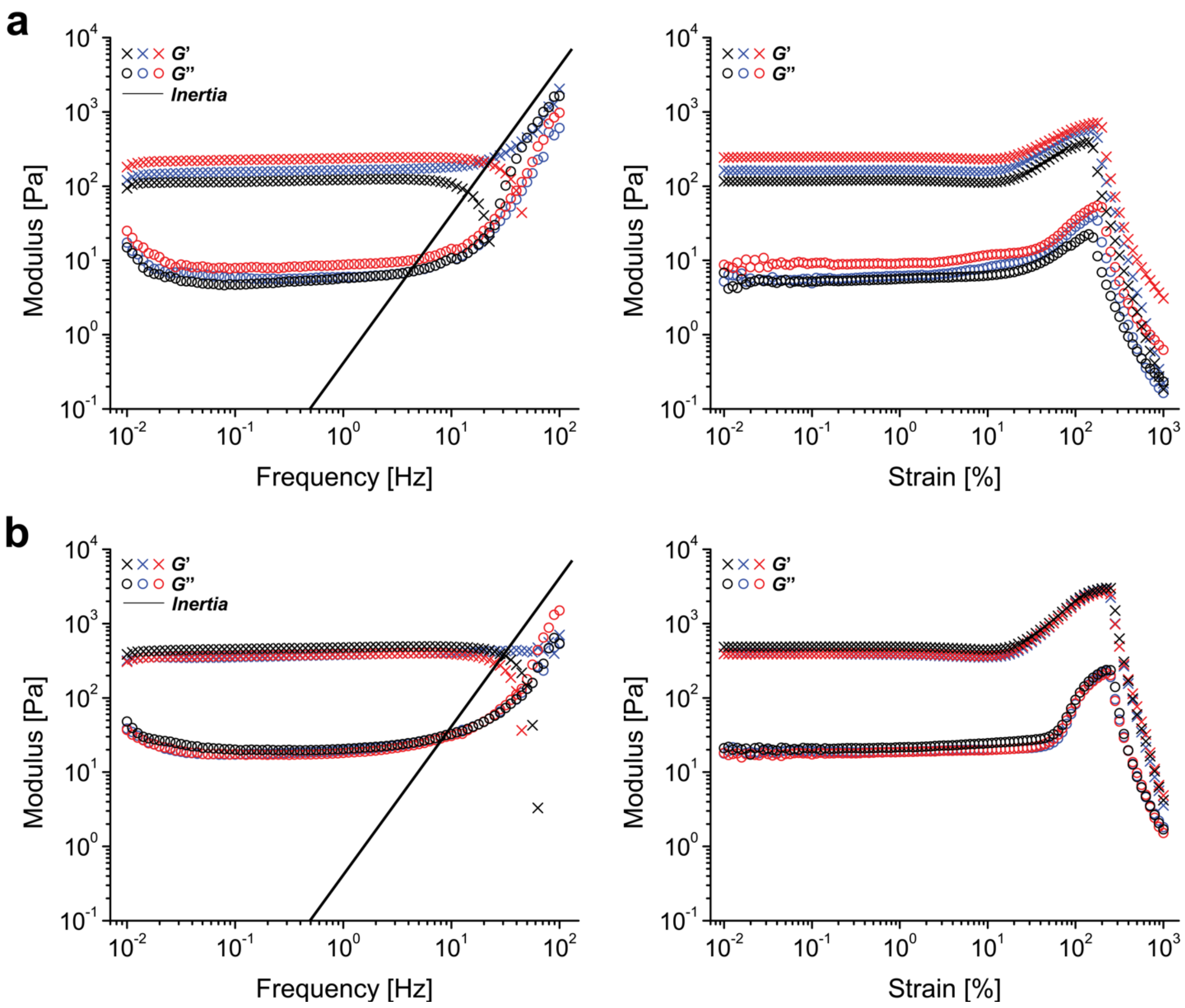

Figure S1 | Macroscopic rheology of fibrin gel samples. Measurement of storage *G*′ and loss *G*′′ moduli as a function of frequency and strain amplitude sweeps for **(a)** 2.5 mg·mL^{-1} and **(b)** 10.0 mg·mL^{-1} fibrin hydrogels. × and ○ symbols represent values for the storage *G*′ and loss *G*′′ moduli, respectively. Red, blue and black symbols represent the results obtained on three separate samples. Frequency and amplitude sweeps were performed with 1% strain and 1 Hz frequency, respectively. The line on the frequency sweep plots represents rheometer inertia. Data points that lie beyond this line are subject to inertial effects produced by the rheometer. Prepared using MATLAB R2019b and Adobe Illustrator CS6.

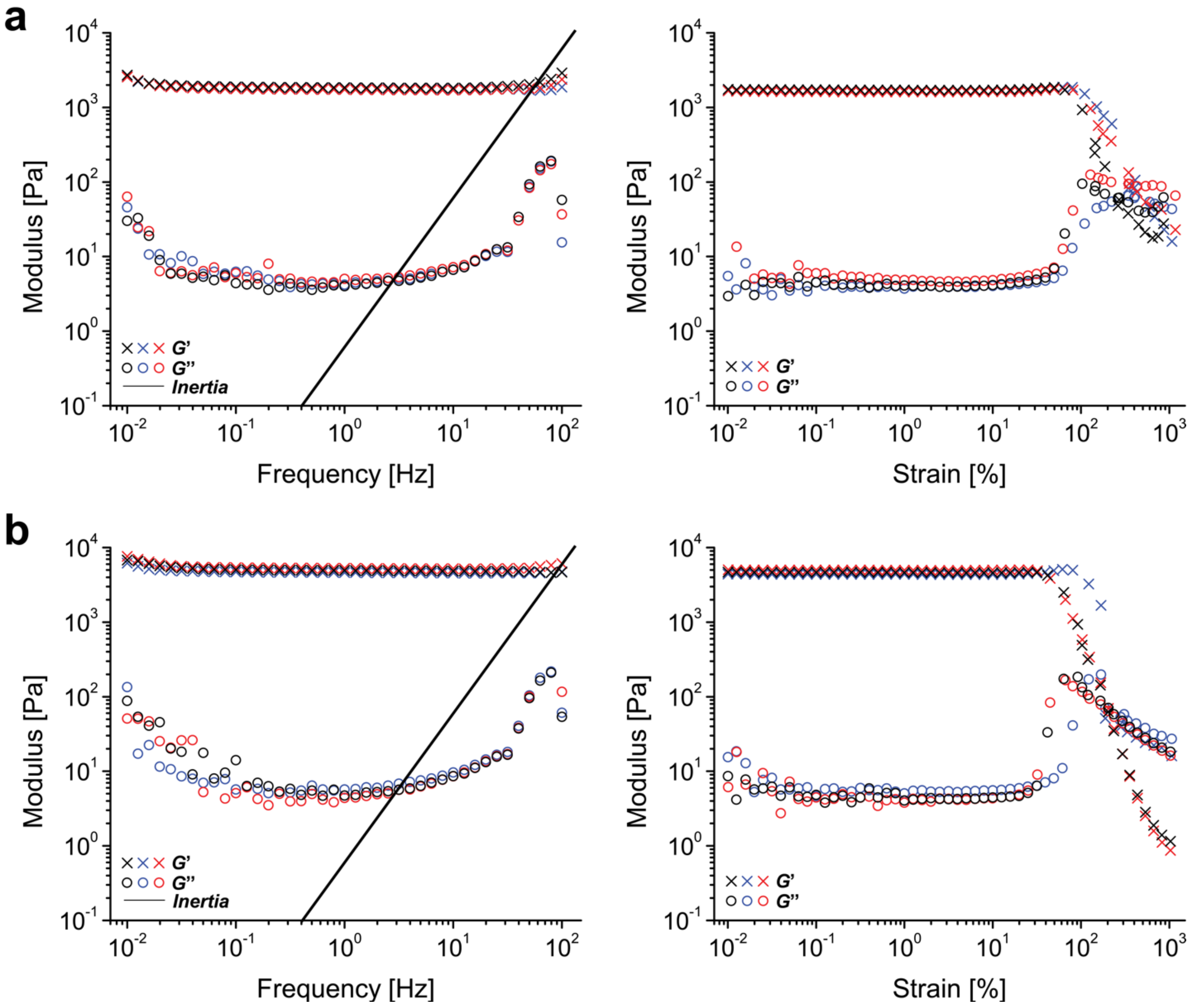

Figure S2 | Macroscopic rheology of PEG (600) DA hydrogels. Measurement of storage *G*′ and loss *G*′′ moduli as a function of frequency and strain amplitude sweeps for **(a)** 6% and **(b)** 7% PEG (600) DA hydrogels. × and ○ symbols represent values for the storage *G*′ and loss *G*′′ moduli, respectively. Red, blue and black symbols represent the results obtained on three separate samples. Frequency and amplitude sweeps were performed with 1% strain and 1 Hz frequency, respectively. The line on the frequency sweep plots represents rheometer inertia. Data points that lie beyond this line are subject to inertial effects produced by the rheometer. Prepared using MATLAB R2019b and Adobe Illustrator CS6.
